# The Effect of Season and Neighbourhood-Built Environment on Home Area Sedentary Behaviour in 9–14 Year Old Children

**DOI:** 10.3390/ijerph18041968

**Published:** 2021-02-18

**Authors:** Larisa Lotoski, Daniel Fuller, Kevin G. Stanley, Daniel Rainham, Nazeem Muhajarine

**Affiliations:** 1 Department of Community Health and Epidemiology, University of Saskatchewan, Saskatoon, SK S7N 5E5, Canada; Larisa.lotoski@usask.ca; 2 School of Human Kinetics and Recreation, Memorial University of Newfoundland, St. John’s, NL A1C 5S7, Canada; dfuller@mun.ca; 3 Saskatchewan Population Health and Evaluation Research Unit, University of Saskatchewan, Saskatoon, SK S7N 5E5, Canada; 4 Department of Computer Science, University of Saskatchewan, Saskatoon, SK S7N 5C9, Canada; kstanley@cs.usask.ca; 5 School of Health and Human Performance, Dalhousie University, Halifax, NS B3H 4R2, Canada; Daniel.Rainham@Dal.Ca

**Keywords:** sedentary behaviour, built environment, season, adolescents, children, physical activity, physical behaviour, sedentary time

## Abstract

There is little understanding of how the built environment shapes activity behaviours in children over different seasons. This study sought to establish how seasonal weather patterns, in a given year in a mid-western Canadian city, affect sedentary time (SED) in youth and how the relationship between season and SED are moderated by the built environment in their home neighbourhood. Families with children aged 9–14 years were recruited from the prairie city of Saskatoon, Canada. Location-specific, device-based SED was captured in children during three timeframes over a one-year period using GPS-paired accelerometers. Multilevel models are presented. Children accumulated significantly greater levels of SED in spring but significantly less SED in the fall months in comparison to the winter months. Children living in neighbourhoods with the highest density of destinations accumulated significantly less SED while in their home area in comparison to their counterparts, and this effect was more pronounced in the spring and summer months. On weekends, the rise in sedentariness within the home area was completely diminished in children living in neighbourhoods with the greatest number of destinations and highest activity friendliness. These results suggested that increasing neighbourhood amenities can lead to a reduced sedentariness of youth, though more so in the warmers months of the year.

## 1. Introduction

The precarious combination of obesity, physical inactivity, and sedentary time (SED) among children requires urgent global attention [1,2], particularly among industrialized countries including those countries with rapidly developing economies [3]. Among 38 countries examined by Tremblay et al. [4], less than half of children and youths (20–39%) met the proposed screen time limit of ≤2 h per day, a marker of physical inactivity and SED. In Canada, children are becoming increasingly overweight and obese [5], and they consistently do not meet recommended guidelines to limit sedentariness (no more than 2 h per day of recreational screen time) [6]. Among Canadian youth aged 5–17 years, 49.3% meet recommended screen time limits and 36% meet PA recommendations [7]. Seventy percent of a youth’s SED consists of screen time. Screen time is considered an adequate surrogate for sedentary behaviour [8].

The results of studies investigating the associations between the built environment and children’s activity behaviours have been mixed. The activity friendliness of children’s home neighbourhoods, such as the presence of parks [9,10,11], open spaces, commercial destinations [9,12], and overall activity friendliness [9,10] can be predictive of physical activity (PA) or SED behaviours. However, two systematic reviews focused on children revealed that many built environment features are only associated with small to moderate increases in PA [13]; furthermore, the construction of neighbourhood infrastructures theorized to promote active lifestyles have shown limited efficacy at increasing PA [14]. A recent evidence synthesis suggested that for children and youth, active transportation behaviours and school environments/settings are associated with increased PA [15].

The winter months are consistently associated with increased sedentary behaviour in children [16,17,18,19,20]. However, the majority of these studies have taken place in regions of the world where average winter temperatures do not drop below −5 °C [16,17,21], have had a lack comprehensive comparisons between all four seasons [21,22,23], or have solely focused on specific weather conditions and seasons [24,25,26]. Furthermore, this existing body of research has primarily focused on mean activity behaviour over the entire study period—as expressed by mean counts per minute [16,18] or mean daily activity behaviour [21]—but have not reported location or time specific physical behaviour (i.e., PA or SED) outcomes. Though a few studies have incorporated analyses of the timing and location of activity behaviours [19,20], we are unaware of any research that has focused on specific locations of sedentary behaviour over longer periods of time among different seasons. There is little understanding of how the built environment shapes sedentary behaviour in children over different seasons.

Canada, the USA, and Northern Europe experience annual seasonal temperature variations of up to 30 °C. Though there is evidence of a relationship between season and activity behaviours in children, measures of seasonal variation are rarely incorporated into studies of the relationship between the built environment and children’s PA behaviours. Of studies that have included season, the intent was to “control” for the timing of data collection [27] rather than to investigate seasonal effects in a more explicit fashion. In a previous study conducted by our research group, 10–14 year old children living in neighbourhoods with varying degrees of destination density and road connectivity demonstrated divergent activity behaviour patterns when exposed to equivalent weather conditions [24]. Destinations are places considered areas of interest for all ages—parks, indoor and outdoor recreation facilities, retail or commercial vendors, schools, libraries, etc. The density of these destinations reflects the abundance of these areas of interest within a single neighbourhood. Again, PA has been the primary outcome of these studies; we cannot extrapolate findings from studies on PA to SED. In this paper, we aimed to fill this knowledge gap.

Children accumulate the most SED while indoors. In a study of Saskatoon 9–15-year-old children, those who spent no time outdoors accumulated an additional 70 more sedentary minutes per day in comparison to those who spend the most time outdoors, and SED decreased in a dose-like manner with increased time spent outdoors [24]. In a review of sedentary behaviour outcomes by Maitland et al., home yard space was the only home attribute measured across all examined studies. The association between yard size and sedentary behaviour outcomes were mixed, resulting in a call for more thorough investigations of home area attributes and sedentary behaviour outcomes [28]. The role of the home-built environment and season on SED in their home property has not been explored.

The authors of this study examined the association between the built environment and sedentary behaviour accumulated in both the indoor and outdoor areas of children’s home properties over a one-year period. As a secondary objective, the analysis examined the effect modification of weather on associations between the home neighbourhood-built environment and home area sedentary behaviour. We hypothesized that children in neighbourhoods with the most things to see, things to do, and overall activity friendliness would accumulate less SED in their home area than their counterparts, but that this difference would diminish during the coldest months of the year.

## 2. Materials and Methods

### 2.1. Study Population and Design

**Figure 1:**
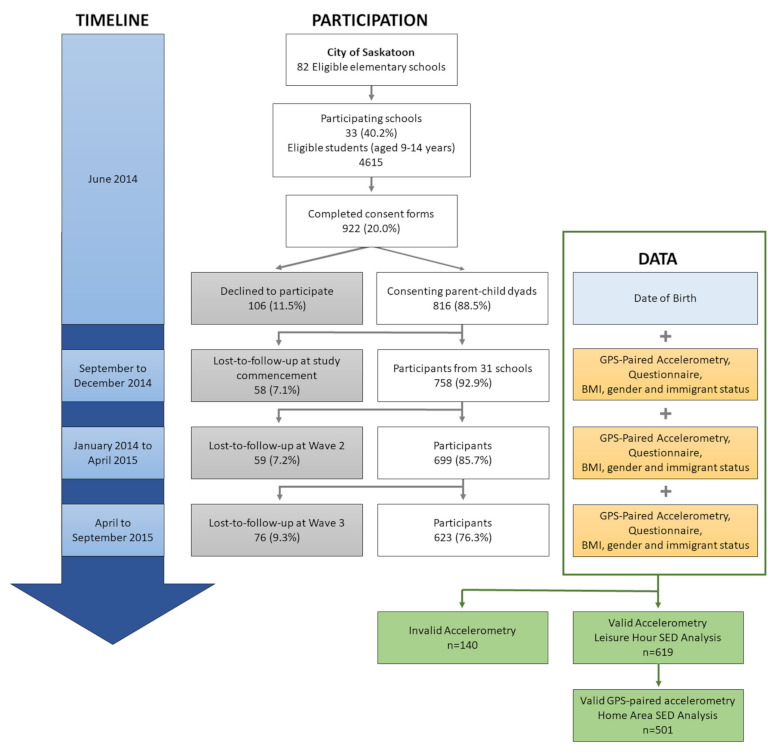
Study timeline, participation and data collection, seasonality and active saskatoon kids (SASK) study. SED: sedentary time.

This study used data from the Seasonality and Active Saskatoon Kids (SASK) study. This study took place in in the mid-sized prairie city of Saskatoon, Canada (52.1579° N, 106.6702° W). The SASK study was a longitudinal cohort study that examined the effects of the neighborhood-built environment on child health through physical activity opportunities across all seasons among children aged 9–14 years. The detailed employed methodology was published previously [29]. In summary, a quota-based sampling method (i.e., grade, gender and school neighbourhood socioeconomic status) was employed to recruit children from a sampling frame that consisted of all 65 residential neighborhoods in Saskatoon. Of the 82 invited schools, 33 (40.2%) participated in the study. From the participating schools, children and their parents were invited to participate in the study through a written informed consent letter disseminated by home classroom teachers. Of the 4615 eligible students in those 33 schools, 922 (20.0%) students and their parents consented. Data were collected at three one-week-long time points over a one-year period: (1) from September to December 2014, (2) from January 2014 to April 2015, and (3) from April to September 2015 (See Figure 1). Children provided up to 21 days of activity behaviour data from three data collection periods lasting seven days each. Limited devices divided amongst study participants prevented us from capturing data within each season for all study participants. This study was approved by the Behavioural Research Ethics Board at the University of Saskatchewan, Canada (Beh #14-83).

### 2.2. Child Anthropometric Measures

Standing height (cm) and weight (kg) were measured using a stadiometer (units: cm) and a digital scale (units: kg) on the day of accelerometry and GPS device deployment. BMI values obtained at the first collection time point were used for all subsequent analyses. BMI was calculated using the World Health Organization (WHO) age- and sex-specific growth reference BMI sample for children and youth aged 5–19 years [30], and it is expressed as *z* scores.

### 2.3. Objective Physical Behaviour Measures

Children’s SED and physical activity behaviours were measured using ActiGraph GT3X accelerometers (ActiGraph Corp., Pensacola, FL, USA) at each of the three time points. Accelerometers were delivered to study participants’ schools, where children were instructed to wear the accelerometer for 8 consecutive days (including sleeping hours) unless entering water. For all participants, accelerometry data acquisition began on the following day at 00:00 h, allowing for 7 complete days of recording. Children were instructed to wear the accelerometer on the posterior to the right iliac crest of the hip [31].

Valid accelerometry data at each collection time point were defined as a minimum daily wear time of 10 h over at least 4 days during a 7-day collection period. Biologically implausible data [32] and non-wear time were defined as >15,000 counts per minute (cpm) and 60 min epochs with <2 min interruptions of continuous 0 s [33], respectively, and they were excluded from the analysis. Activity level cut points were defined as follows: SED ≤ 150 counts per minute (cpm), light physical activity (LPA; 150–1951 cpm), and moderate-to-vigorous physical activity (MVPA; from 1952 to ≥5724 cpm, excluding >15,000 cpm biologically implausible data) [34]. SED, LPA, and MVPA described in this study explicitly refer to physical behaviours accumulated only within a child’s home property buffer. Physical behaviours accumulated beyond a child’s home property were not considered in the presented analyses.

### 2.4. Sedentary Behaviour Location Context

Children’s home area physical behaviours were derived from location data collected using the GPS receiver loggers. Belts equipped with accelerometers were housed with a Qstarz BT-1000XT Travel Recorder GPS logger (Qstarz International Co. Ltd., Taipei, Taiwan). GPS-derived latitude and longitude were averaged over one-minute epochs to allow for pairing with accelerometry data. A child’s residential location was identified using parcel (polygon) shapefiles obtained from the City of Saskatoon (April–November 2017). The physical behaviour measures utilized in this study included accelerometry data points that fell within a 20 m buffer of a child’s home.

### 2.5. Neighbourhood-Built Environments

Saskatoon’s six developing (as of time of data collection in 2014) and sixty established neighbourhoods were defined by municipal boundaries. A child’s home neighbourhood was defined as the neighbourhood in which a child’s home property fell. Neighbourhood environment characteristics were collected for Saskatoon’s 66 neighbourhoods using two validated audit tools, the Irvine Minnesota Inventory (IMI) [35] and Neighbourhood Active Living Potential (NALP) [36] tools in the summer of 2012 and July–August 2014. The IMI is “an extensive audit tool aimed at measuring a broad range of built environment features that may be linked to active living,” [37] and is made up of 160 items. The NALP is a 22-item tool measuring domains thought to promote active living. NALP has been shown as a reliable environment measure in the context of Saskatoon [38]. Both the IMI and NALP utilize dimension scores for latent neighbourhood-scale built environment attributes [39,40] and consider the theoretical barriers and facilitators of children’s physical movement. IMI dimension scores were obtained by auditing a random selection of a neighbourhood’s street segments (10%). NALP dimension scores were obtained by walking an approximately 4–5 km continuous segment within a neighbourhood. Continuous segments were created by randomly selecting 20% of all street segments within a neighbourhood and manually connecting them together to create a walking route.

In this analysis, NALP cumulative scores, NALP dimension scores, IMI cumulative scores, and IMI inventory scores were employed in multivariable analysis to predict SED behaviour.

### 2.6. Season

Seasons were defined using northern meteorological seasons, where winter, spring, summer, and fall are defined by calendar date, from 1 December to 28/29 February, 1 March to 31 May, 1 June to 31 August, and 1 September to 30 November, respectively [41]. The date on which GPS-paired accelerometry were collected determined the season assigned to the data.

### 2.7. Data Acquisition, Cleaning and Analysis

Data cleaning, manipulation, analysis, and visualization were performed in R [42] and R Studio [43], unless otherwise stated. Accelerometer-generated data were analyzed at 1 min epochs using the ActiLife 6 data analysis software (Version 6.11.4, ActiGraph Corp., Pensacola, FL, USA).

To allow for pairing to 1 min epoch accelerometry data, GPS data acquired at 1 sec epochs were aggregated to 1 min epochs using Python (Version 2.7.11) [44], by calculating the median location from latitude and longitude coordinates. Velocities > 100 km/hour and GPS coordinates falling outside of Saskatoon city limits were excluded from the analysis using shapefiles obtained from the city of Saskatoon (April–November 2017). GPS-paired accelerometry data were considered valid if participants accumulated a minimum of 8 h per day over 3 days per collection time point. QGIS (Version 2.18.9, QGIS Geographic Information System. QGIS Association. http://www.qgis.org (accessed on 10 February 2021)) was used for the analysis of location data [45]. The home area was defined as GPS data points falling within 20 m of the centroid of a participant’s residential parcel.

SED was described using univariate models and multiple variable multilevel modeling. Univariate models included (level 1) individual-level repeated measures: season, LPA, and MVPA accumulated on the same day and location or time period as the SED outcome measure. Multilevel models describing the prediction of SED including (level 1) individual level repeated measures, (level 2) individual measures, and (level 3) neighbourhood level measures. Level 2 independent variables included gender, age, BMI, highest annual household income reported during the study, and newcomer (living within Canada for less than 2 years). Level 3 independent variables included NALP dimension scores (aesthetic factors, density of destination, safety, and universal accessibility), IMI dimension scores (density of destination, attractiveness, pedestrian accessibility, safety from crime, safety from traffic), NALP and IMI cumulative scores, and combined NALP and IMI scores. Multivariable multilevel models were built using a backwards step-wise approach, first establishing level 1 main effects and then level 2 main effects. Only level 1 and 2 variables demonstrating a significant prediction (*p* < 0.05) of SED and improved model fit were included. Level 3 variables were added to each model one at a time, where significance and model fit were assessed using Akaike’s information criterion (AIC) and Bayesian information criterion (BIC). Main effect models were tested for confounding at each step of the model building process. The annual household income variable category “Unknown” included those who actively chose not to answer, did not know their annual household income, or did not provide an answer.

## 3. Results

### 3.1. Sample Characteristics

Of the 816 children who consented to participate in the study, 758 children attended at least one data collection time point. At the first (September–December 2014), second (January–April 2015), and third (April–June 2015) data collection time points, 58 (7.1%), 59 (7.2%), and 76 (9.3%) of the original consenting population students were lost to follow up (either absent, had moved to a different school or province, or declined to participate further), respectively (Figure 1). Of study participants, 519, 411, and 301 participants contributed valid accelerometry data during the first, second, and third collection time points, respectively. Of these participants, 613 participants recorded at least 8 h of GPS data per day for at least 3 days per time point. During the first, second, and third collection time points 307 (40.5%), 345 (45.5%), and 247 (32.6%) of participants contributed both valid accelerometry and GPS data, respectively. The final study population included 455 participants.

As shown in Table 1, our analysis study population was over-represented by female children in comparison to the 2016 Census Profiles for Saskatoon and Canada [46]. Both study populations examined in this study comprised of fewer children with normal weight and a greater number of children who were overweight in comparison to the Canadian population. One-quarter of participating families either refused to report or did not know their annual household income (Table 1).

### 3.2. Children’s Home Area Sedentary Time

Participating children spent 31.1% of their day within 20 m of their home property, and 56.8% of all participant activity minutes fell within a participant’s home neighbourhood. On weekends, children spent more than half their day at home (59.9%). Multilevel models were developed to assess the influence of demographic characteristics, season and built environment feature on children’s home area SED time.

#### 3.2.1. Individual-Level Factors Associated with Children’s Home Area Sedentary Time

When children were within their home area, they accumulated significantly more SED if they were male, older, and had immigrated to Canada within the past two years. Seasonality effects on home area SED were evident. Children had 12 fewer minutes of home area SED per day in the fall months (vs winter); they had a modest additional 7 min of home area SED per day during the spring months compared to that in the winter months.

#### 3.2.2. Neighbourhood-Level Factors Associated with Children’s Home Area Sedentary Time

Children in neighbourhoods with a higher density of destinations were significantly less likely to engage in home-based SED compared to children in neighbourhoods with a lower density of destinations. Additionally, children in neighbourhoods with the highest density of destinations had no significant increase in home area SED on weekends in comparison to those in neighbourhoods with a low density of destinations. In contrast, children living in neighbourhoods with the highest level of safety from crime or overall activity friendliness had significantly more SED (Table A1).

#### 3.2.3. Neighbourhood Factors Moderates Seasonality Effects, Household Income on Children’s Home Area Sedentary Time

**Figure 2:**
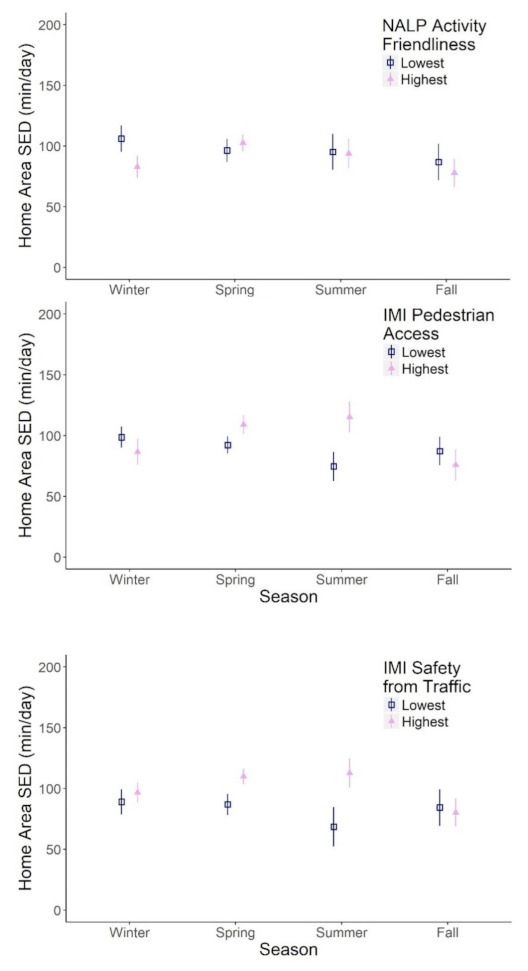
The effect of season on home area sedentary time (SED) in children is moderated by home neighbourhood built environment. The presented predicted effects are derived from multilevel models level 3 non-main effects interaction terms presented in Table A1 (model 1: season*NALP (Neighbourhood Active Living Potential) activity friendliness; model 2: season*IMI (Irvine Minnesota Inventory) pedestrian access; model 3: IMI safety from traffic). 95% CI are shown as vertical bars.

Neighbourhood activity friendliness, pedestrian access, and safety from traffic significantly moderated the effect of season on home area SED. In the winter months, children in neighbourhoods with the lowest level of activity friendliness and pedestrian access were significantly more sedentary in their home property than children in neighbourhoods with higher activity friendliness and pedestrian access. This relationship was diminished and even reversed in some instances in the spring and summer months. In the summer months but not the winter months, children in the safest neighbourhoods were significantly more sedentary than their counterparts (Figure 2).

**Figure 3:**
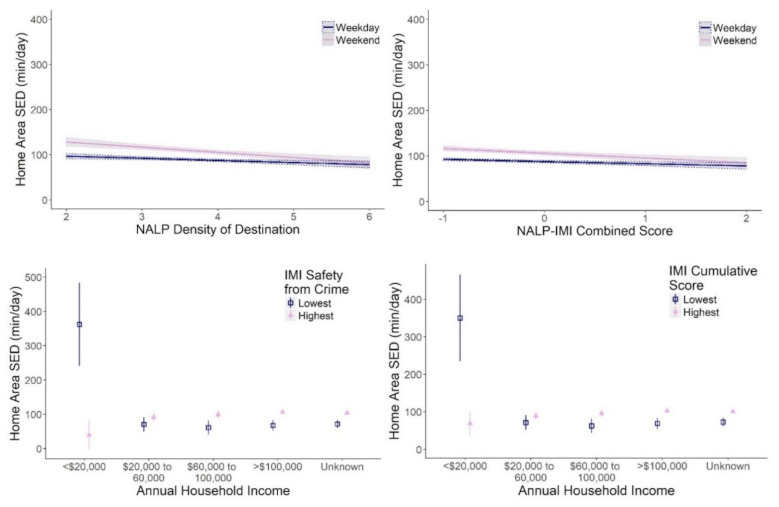
The effect of annual household income and weekends (vs. weekdays) on home area sedentary time (SED) in children are moderated by the built-environment characteristics of the home neighbourhood. Shown predicted effects are derived from the multilevel models presented in Appendix A, Table A1 (Model 4: weekday*NALP density of destination; Model 7: weekday*NALP–IMI combined score; Model 5: income*IMI safety from crime; Model 6: income*IMI cumulative score). 95% CIs are shown as vertical bars or grey ribbons.

Children in neighbourhoods with a higher degree of physical safety (IMI safety from crime) and overall activity friendliness (IMI cumulative score) had significantly more home area SED time than children in neighbourhoods with a lower degree of safety from crime and activity friendliness (Table 2). The effect of household income on home area SED time was moderated by both neighbourhood safety and activity friendliness. Children in low-income households (annual income <$20,000) were significantly likely to have accumulated more home area SED time than children in high-income households; this was particularly true for children in neighbourhoods with the lowest safety from crime and activity friendliness (Figure 3).

As shown in Figure 3 (Table A1), multiple neighbourhood-built environment attributes significantly predicted a child’s home area SED. Children in neighbourhoods with the highest NALP density of destination and NALP–IMI combined scores had significantly lower SED times. Neighbourhood destination density and NALP–IMI combined scores moderated the effects on home area SED in weekends. Children in neighbourhoods with the highest density of destinations and overall activity friendliness had no increase in home area SED on the weekend compared to children in neighbourhoods with a lower density of destinations or activity friendliness.

Models were established by testing all variables listed: level 1 (home area LPA (min/day), home area MVPA (min/day), season, and weekday), level 2 (age, gender, BMI, income, and newcomer to Canada immigrant status) and level 3 (NALP dimension scores: density of destinations, activity friendliness, safety and universal accessibility; NALP cumulative score; IMI dimension scores: density of destinations, pedestrian access, attractiveness, safety from crime, safety from traffic; and IMI cumulative score). The final presented models only included variables that contributed to the most parsimonious models.

## 4. Discussion

This study sought to better understand the complex relationship between home area SED and demographic factors, social environments, and physical environments (including seasons) in children 9–14 years of age year-round. The research presented here brings evidence that physical environments, constrained by time and geographic location (home area) where children spend the majority of their day, have common individual-level but divergent neighbourhood-level predictors of SED.

### 4.1. Sedentary Time and Individual Level Demographic Factors

Within their home environment, older children accumulated greater levels of SED in comparison to younger children. While males were significantly more sedentary than females within their home areas, females demonstrated a greater increase in sedentariness with increased age. The phenomenon of decreased PA [50,51,52,53,54] and increased sedentariness [55,56] with increased chronologic age and biological maturation has been reported elsewhere. Physical behaviour trajectories may be partially driven by differences in the timing of biological maturation, physical literacy [54], and independent mobility [57,58] experienced between genders. Additionally, female adolescents in this study and elsewhere [59,60,61,62] reported watching television and using communication-based screen time (phones) for longer durations than their male counterparts.

### 4.2. Sedentary Time and Home Neighbourhood-Built Environment

In this study, specific built environment features were associated with small but discernible changes in home area SED. Children in neighbourhoods with the highest density of destinations accumulated six fewer minutes of SED/day in their home area in comparison to their counterparts. Increased home-based SED behaviours on weekends were moderated by neighbourhoods with a large number of destinations and activity friendliness. Children living in neighbourhoods with the lowest density of destinations and overall activity friendliness were significantly more sedentary on weekends (vs weekdays) when in their home area. This weekend–weekday difference was not visible in children living in neighbourhoods with the highest level of destinations and overall activity friendliness. These results suggested that increasing retail and recreational destinations through infill and new developments has the potential to shift children’s health through the reduction of SED.

Children in neighbourhoods with higher levels of social disorder and overall activity friendliness accumulated greater amounts of home area SED. Children living in low-income neighbourhoods were found to be able to walk or bike significant greater distances on their own [63], accumulate the greatest levels of MVPA [64], and be afforded greater levels of independent mobility in comparison to their counterparts, even after parental attitudes towards independent mobility were adjusted for [58]. Findings similar to ours were described by Gauvin et al. [39], but this was in a population of Canadian adults. Higher levels of NALP safety, in comparison to average safety, were associated with a lower likelihood of walking in adults [39]. Children of this age group, 9–14 years, are still under the supervision of their parents, and concerns for safety for their children might have restricted their independent mobility and play outside the home, even while still on one’s home property. While speculative, it is possible that children living in Saskatoon’s least safe neighbourhoods are offered greater levels of free unsupervised play out of necessity. Other studies have shown that children living in the lowest socioeconomic status areas are allowed to walk or bike significantly greater distances on their own [63] and accumulated the greatest levels of MVPA [64] in comparison to their counterparts. Similarly, children living in low-income neighbourhoods of Toronto, Ontario were afforded greater levels of independent mobility, even after parental attitudes towards independent mobility were adjusted for [58].

### 4.3. Seasonal Differences in Sedentary Time Outcomes

As expected, the effect of seasons on home area SED was evident in this study. Children had lower levels of home area SED in the fall season compared to the winter season. We found greater accumulated home area SED time during spring (vs winter); this was in contrast to other research on seasonal changes in children’s activity behaviours [16,17,18,19,20,21]. Additionally, the effect of season was moderated by the neighbourhood-built environment. Among the limited studies simultaneously considering season and the built environment, moderating effects have been reported. For example, in a cohort of children aged 11–12 years living in Cyprus, seasonal differences in step-counts were moderated by residents of urban vs. rural environments [65]. Similarly, in a Kingston, ON, based study of 10–13-year-old youth, neighbourhood walkability was associated with greater levels of active transport. The difference in active transport trips between the least and most walkable neighbourhoods was greatest in the spring months and diminished in the winter months [66]. These results suggested that alterations to the built environment will result in greater physical behaviour change in the warmest months of the year. However, it is unclear if interventions targeting built-environment features in winter conditions could reduce children’s home area SED in the colder months of the year (e.g., improved snow and ice clearing, as well as public warming shelters).

### 4.4. Strengths and Limitations

The strengths of this study included the device-based measures of children’s SED and their corresponding geographic locations, survey data exploring multiple demographic factors, and neighbourhood-level built environment data over four seasons in one Canadian city [67]. Through the use of multilevel models, individual-, social-, and environment-level characteristics were simultaneously considered. A second major contribution of this study was the simultaneous consideration of waking physical behaviours (LP and, MVPA) as they occurred in time and place. Participants provided up to 21 days of device-based activity data over an entire year, allowing for seasonal context to be included in our analyses, all of which provided novel insights into what predicts home area SED in children.

Neighbourhood-level audits utilizing the NALP and IMI audit tools were conducted during the summer months and pleasant weather conditions with no ground cover. Natural light exposure, common winter (snow and ice ground cover), shoulder season (leaves, snowmelt, and street or sidewalk drainage issues) physical environment attributes were not considered in this analysis. Non-wear time was not included in our presented models. Our measure of socioeconomic status was limited to participating families’ annual household income and no other commonly used measures (parent or maternal highest education attainment, parent occupation(s), household dynamic, etc.). This study was limited to children’s physical behaviours within their home properties and not a representation of physical behaviours over their entire day. This study primarily occurred during the regular school year (September–June), resulting in a lower proportion of data being collected during the summer months.

## 5. Conclusions

The simultaneous examination of both season and the built environment over one year was a unique feature of this study. The focus on one aspect of SED, home area SED, and the consideration of light and moderate-to-vigorous PA were strengths and not commonplace in previous research. Our study highlights the significant increase in sedentariness as children age, emphasizing the critical need to understand the multi-level and all-season determinants of children’s physical behaviour during this critical period of development. The results provide important new insights about interactions between home area SED, neighbourhood factors, and seasons.

## Figures and Tables

**Table 1:** Study population characteristics.

	Consenting Study Participants *	Valid Accelerometry Data	Valid Accelerometry and GPS Data within City Limits	City of Saskatoon	Canada
	***n*** **(%)**	***n*** **(%)**	***n*** **(%)**	***n*** **(%)**	***n*** **(%)**
**Total Sample**	758	619	455	246,376	35,151,728
Gender **					
Male	345 (45.5)	256 (41.5)	172 (37.8)	7050 (51.4)	985,200 (51.2)
Female	413 (54.5)	361 (58.5)	283 (62.2)	6655 (48.6)	937,445 (48.8)
**Age**					
9	33 (4.4)	29 (4.7)	17 (3.7)		
10	236 (31.1)	199 (32.3)	158 (34.7)		
11	234 (30.9)	195 (31.6)	147 (32.3)		
12	151 (19.9)	118 (19.1)	78 (17.1)		
13–14	104 (13.8)	76 (12.3)	55 (12.0)		
10–14	-	-	-	13,705	1,922,645
**Body Mass Index ^†^**					
Neither overweight nor obese	-	-	-	-	4,604,500 (76.0)
Underweight	9 (1.2)	6 (1.0)	1 (0.2)	5.3%	
Normal Weight	465 (61.3)	382 (61.9)	285 (62.6)	73.4%	
Overweight	173 (22.8)	145 (23.5)	111 (24.4)		980,300 (16.2)
Obese	111 (14.6)	84 (13.6)	58 (12.7)		477,500 (7.9)
Overweight or obese				21.30%	
**Immigrant Status** ^**‡**^					
Newcomer	95 (12.6)	71 (11.5)	52 (11.4)	4160 (7.5)	216,320 (3.6)
Non-Newcomer	660 (87.4)	545 (88.5)	402 (88.4)	51,315 (92.5)	5,839,565 (96.4)
**Annual Household Income**					
<$20,000	14 (1.8)	12 (1.9)	5 (1.1)	7380 (5.4)	1,369,620 (7.4)
$20,000–$60,000	110 (14.5)	86 (13.9)	38 (8.4)	29,445 (21.7)	4,623,370 (24.8)
$60,000–$100,000	121 (16.0)	104 (16.9)	49 (10.8)	24,340 (17.9)	3,517,155 (18.9)
>$100,000	295 (38.9)	258 (41.8)	157 (34.5)	74,810 (55.0)	9,123,845 (49.0)
Unknown	218 (28.8)	157 (25.4)	206 (45.3)		
***Season***					
Winter	626 (28.9)	415 (29.6)	277 (33.0)		
Spring	635 (29.3)	348 (24.9)	291 (34.6)		
Summer	256 (11.8)	119 (8.5)	96 (11.4)		
Fall	651 (30)	518 (37.0)	176 (21.0)		

* Excludes consenting participants that did not participate in any data collection time points. ** From the 2016 Census Profile for Canada (country) and Saskatoon, CY (census subdivision), Saskatchewan (table) [46]. ^†^ Body Mass Index: study population BMI was calculated using the World Health Organization (WHO) Age- and sex-specific growth reference BMI sample for 5–19-year-olds [30]. BMI for grades 5–8 children living in the City of Saskatoon was obtained from 2010/2011 Student Health Survey [47] and for children living within Canada from the 2015 Canadian Community Health Survey—Nutrition [48]. ^‡^ Immigrant status: study population newcomer status were children who reported living within Canada for less than two years. The city of Saskatoon and Canada population newcomers were children aged 0–14 who immigrated to Canada between 2011 and 2016, as reported in the 2016 Census [49].

**Table 2:** Factors predicting home area sedentary time in 9–14-year-old children: univariate and individual level main effect models.

	Model 1 Beta (95%CI)	Model 2 Beta (95%CI)	Model 3 Beta (95%CI)	Model 4 Beta (95%CI)
**Sample Size (*n*)**	420	420	420	420
**Constant**	−7.6 (−63.8, 48.6)	−83.2 (−148.0, −18.1)	−31.2 (−82.8, 20.4)	−31.0 (−82.7, 20.6)
**Level 1 Variables**				
LPA	0.8 (0.8, 0.9)	0.8 (0.8, 0.9)	0.8 (0.8, 0.9)	0.8 (0.81, 0.9)
MVPA	−0.4 (−0.5, −0.3)	−0.4 (−0.5, −0.3)	−0.4 (−0.5, −0.3)	−0.4 (−0.5, −0.3)
Season				
Spring	6.9 (1.7, 12.1)	6.8 (1.5, 12.0)	6.8 (1.5, 12.0)	6.8 (1.6, 12.1)
Summer	1.4 (−6.3, 9.2)	0.8 (−6.9, 8.5)	0.8 (−6.9, 8.5)	1.4 (−6.4, 9.1)
Fall	−11.6 (−18.3, −4.8)	−11.6 (−18.4, −4.9)	−11.6 (−18.4, −4.9)	−11.5 (−18.3, −4.7)
Weekend Day	19.8 (15.7, 23.9)	19.8 (15.7, 24.0)	19.8 (15.7, 24)	19.8 (15.7, 23.9)
**Level 2 Variables**				
Age	7.7 (4.5, 10.9)	7.6 (4.5, 10.8)	7.6 (4.45, 10.8)	7.7 (4.5, 10.9)
Gender	−11.2 (−18.6, −3.9)	−11.1 (−18.4, −3.8)	−11.1 (−18.4, −3.8)	−11.2 (−18.5, −3.8)
BMI				
Underweight	−24.1 (−89.1, 40.9)	−27.1 (−91.9, 37.7)	−27.10 (−91.9, 37.7)	−24.3 (−89.2, 40.6)
Overweight	6.5 (−1.9, 14.9)	6.9 (−1.5, 15.2)	6.85 (−1.54, 15.2)	6.8 (−1.6, 15.2)
Obese	14.8 (3.6, 26.1)	15.0 (3.8, 26.3)	15.00 (3.78, 26.3)	14.9 (3.7, 26.2)
Income				
$20,000–$60,000	−34.3 (−69.5, 0.9)	−33.0 (−68.2, 2.2)	−33.0 (−68.2, 2.2)	−34.2 (−69.3, 1.02)
$60,000–$100,000	−30.9 (−66.0, 4.2)	−30.6 (−65.6, 4.5)	−30.6 (−65.7, 4.5)	−31.3 (−66.4, 3.76)
>$100,000	−22.9 (−56.8, 11.1)	−23.4 (−57.4, 10.5)	−23.4 (−57.4, 10.5)	−23.7 (−57.7, 10.2)
Unknown	−24.6 (−58.3, 9.0)	−24.6 (−58.1, 9.0)	−24.6 (−58.1, 9.0)	−25.3 (−58.8, 8.3)
Newcomer to Canada ^‡^	16.1 (4.25, 28.0)	15.5 (3.7, 27.4)	15.5 (3.7, 27.4)	16.1 (4.3, 27.9)
**Level 3 Variables**				
NALP Density of Destination	−6.0 (−12.0, −0.1)			
IMI Safety from Crime		6.2 (1.4, 10.9)		
IMI Cumulative Score			6.7 (1.6, 11.8)	
NALP−IMI Combined Score				−6.1 (−11.3, −0.8)

^‡^ Reference categories: season—winter; gender—male; body mass index—normal weight; income—<$20,000. Abbreviations: BMI—body mass index; Income—annual household income; LPA—home area light physical activity; MVPA—home area moderate-to-vigorous physical activity; Newcomer to Canada—living in Canada for less than two years.

## Data Availability

The data presented in this study are available on request from the corresponding author. The data are not publicly available due to the identifiable nature of home area geographical data.

## Appendix A

**Table A1:** Factors predicting home area sedentary time in 9–14-year-old children: main effect models with interaction terms.

	Model 1 Beta (95% CI)	Model 2 Beta (95% CI)	Model 3 Beta (95%CI)	Model 4 Beta (95%CI)	Model 5 Beta (95%CI)	Model 6 Beta (95%CI)	Model 7 Beta (95%CI)
**Sample Size (*n*)**	420	420	420	420	420	420	420
**Constant**	3.8 (−63.2, 70.8)	1.6 (−81.5, 84.7)	−42.0 (−109.0, 24.7)	−30.5 (−88.0, 27.0)	563.0 (63.3, 1060.0)	−8.3 (−624.0, 45.7)	−30.2 (−81.7, 21.4)
**Level 1 Variables**							
LPA	0.8 (0.8, 0.9)	0.8 (0.8, 0.9)	0.8 (0.8, 0.9)	1.0 (0.9, 1.2)	0.6 (0.3, 0.8)	0.8 (0.8, 0.8)	0.8 (0.8, 0.9)
MVPA	−0.4 (−0.5, −0.3)	−0.4 (−0.5, 0.3)	−0.4 (−0.5, −0.3)	−0.4 (−0.5, −0.3)	−0.4 (−0.5, −0.3)	−0.4 (−0.5, −0.3)	−0.4 (−0.5, −0.3)
Season							
Spring	−38.9 (−80.6, 2.8)	−64.4	−17.4 (−54.9, 20.0)	6.6 (1.4, 11.9)	6.6 (1.3, 11.8)	6.6 (1.3, 11.8)	6.6 (1.4, 11.9)
Summer	−32.7 (−88.7, 23.3)	−129.7 (−210.0, −49.4)	−57.3 (−115.0, 0.7)	0.8 (−6.9, 8.5)	0.3 (−7.4, 8.0)	0.3 (−7.4, 8.0)	0.5 (−7.2, 8.2)
Fall	−33.5 (−87.2, 20.2)	−12.9	7.2 (−43.6, 57.9)	−11.7 (−18.5, −5.0)	−11.7 (−18.4, −4.9)	−11.7 (−18.4, −4.9)	−11.7 (−18.5, −5.0)
Weekend Day	19.7 (15.6, 23.9)	19.8 (15.7, 23.9)	19.7 (15.6, 23.9)	44.8 (24.9, 64.7)	20.1 (16.0, 24.2)	20.1 (16.0, 24.2)	18.3 (14.0, 22.7)
**Level 2 Variables**							
Age	7.8 (4.6, 11.0)	7.7 (4.5, 10.9)	7.8 (4.6, 11.1)	7.8 (4.6, 11.0)	7.5 (4.3, 10.7)	7.5 (4.3, 10.7)	7.7 (4.5, 10.9)
Gender	−11.2 (−18.6, −3.9)	−11.1	−11.4 (−18.8, −4.0)	−10.9 (−18.2, −3.5)	−11.4 (−18.8, −4.1)	−11.4 (−18.8, −4.1)	−10.8 (−18.1, −3.5)
BMI							
Underweight	−27.6 (−92.7, 37.5)	−28.9	−29.1 (−94.6, 36.3)	−24.1 (−89.0, 40.8)	−26.3 (−90.7, 38.0)	−26.3 (−90.7, 38.0)	−24.1 (−88.9, 40.6)
Overweight	6.1 (−2.3, 14.5)	5.9 (−2.6, 14.3)	6.2 (−2.2, 14.7)	6.8 (−1.6, 15.1)	8.3 (−0.1, 16.7)	8.3 (−0.1, 16.7)	6.9 (−1.4, 15.3)
Obese	15.0 (3.6, 26.3)	14.3	15.3 (3.9, 26.7)	14.8 (3.6, 26.1)	15.7 (4.5, 27.0)	15.7 (4.5, 27.0)	14.7 (3.5, 25.9)
Income							
$20,000–$60,000	−34.3 (−69.6, 1.0)	−36.4	−35.9 (−71.4, −0.5)	−34.4 (−69.5, 0.8)	−635.0 (−1140.0, −129.0)	−54.5 (−93.2, −15.9)	−34.1 (−69.2, 1.0)
$60,000–$100,000	−30.9 (−66.1, 4.3)	−33.8 (−69.3, 1.7)	−33.3 (−68.6, 2.1)	−30.4 (−65.5, 4.7)	−663.0 (−1170.0, −158.0)	−51.5 (−89.0, −13.0)	−30.9 (−65.9, 4.1)
>$100,000	−22.00 (−56.1, 12.2)	−25.1	−24.8 (−59.1, 9.5)	−22.9 (−56.8, 11.1)	−657.0 (−1160.0, −154.0)	−44.8 (−82.3, −7.2)	−23.6 (−57.5, 10.2)
Unknown	−23.90 (−57.6, 9.85)	−26.60 (−60.6, 7.35)	−26.00 (−59.9, 7.82)	−24.50 (−58, 9.11)	−646.00 (−1150, −145)	−45.20 (−82.4, −8.05)	−25.00 (−58.5, 8.54)
Newcomer to Canada ^‡^	16.10 (4.24, 28)	15.5	16.10 (4.18, 28.1)	16.20 (4.35, 28.0)	16.10 (4.31, 27.9)	16.10 (4.31, 27.9)	16.20 (4.38, 28.0)
**Level 3 Variables**							
NALP Activity Friendliness	−9.65 (−21.6, 2.32)						
IMI Pedestrian Access		−5.81 (−18.9, 7.33)					
IMI Safety from Traffic			1.9 (−4.9, 8.8)				
NALP Density of Destination				−0.2 (−7.0, 6.6)			
IMI Safety from Crime					−67.5 (−126.0, −9.4)		
IMI Cumulative Score						−73.5 (−137.0, −10.3)	
NALP−IMI Combined Score							−1.1 (−7.1, 4.9)
**Interactions Terms**							
Season*NALP Activity Friendliness ⱡ							
Spring*Activity Friendliness	12.2 (1.2, 23.2)						
Summer*Activity Friendliness	9.1 (−5.9, 24.1)						
Fall*Activity Friendliness	5.9 (−8.2, 20.1)						
Season*IMI Pedestrian Access ⱡ							
Spring*Pedestrian Access		13.8 (2.1, 25.5)					
Summer*Pedestrian Access		25.1 (9.7, 40.6)					
Fall*Pedestrian Access		0.4 (−14.3, 15.0)					
Season*IMI Safety from Traffic ⱡ							
Spring*Safety from Traffic			3.8 (−2.1, 9.7)				
Summer*Safety from Traffic			9.2 (0.1, 18.2)				
Fall*Safety from Traffic			−3.0 (−10.9, 5.0)				
LPA*NALP Density				−0.1 (−0.1, 0.0)			
of Destinations							
Weekend Day*NALP^‡^				−6.7 (−12.0, −1.4)			
Density of Destination							
LPA*IMI Safety from Crime					0.0 (0.0, 0.0)		
Income*IMI Safety from Crime ⱡ							
$20–60,000*Safety from Crime					68.7 (10.9, 126.0)		
$60–100,000*Safety from Crime					72.3 (14.7, 130.0)		
>$100,000*Safety from Crime					72.4 (15.0, 130.0)		
Unknown*Safety from Crime					71.0 (13.9, 128.0)		
LPA*IMI Cumulative Score						0.0 (0.0, 0.1)	
Income*IMI Cumulative Score ⱡ							
$20–60,000*IMI Score						74.8 (11.8, 138.0)	
$60–100,000*IMI Score						78.7 (16.0, 141.0)	
>$100,000*IMI Score						78.8 (16.3, 141.0)	
Unknown * IMI Score						77.4 (15.2, 140.0)	
LPA * NALP−IMI Combined Score							−0.0 (−0.1, 0.0)
Weekend Day*NALP−IMI Combined Score ⱡ							−5.5 (−10.2, −0.9)

^‡^ Reference categories: season—winter; gender—male; body mass index—normal weight; income—<$20,000. Abbreviations: BMI—body mass index; CI—confidence intervals; Income—annual household income; LPA—leisure hour light physical activity (min/day); MVPA—leisure hour moderate-to-vigorous physical activity (min/day); Newcomer to Canada—living in Canada for less than two years. Final main effect models were established using all significant level 1 (home area LPA (min/day), home area MVPA (min/day), season, and weekday), level 2 (age, gender, BMI, income, and newcomer to Canada immigrant status), and level 3 variables (NALP dimension scores: density of destinations, activity friendliness, safety, and universal accessibility; NALP cumulative score; IMI dimension scores: density of destinations, pedestrian access, attractiveness, safety from crime, and safety from traffic; and IMI cumulative score). The final presented models only included those that contributed to the most parsimonious models.

## References

[1] Egger G., Swinburn B. (1997). An “ecological” approach to the obesity pandemic. BMJ.

[2] Kohl H.W., Craig C.L., Lambert E.V., Inoue S., Alkandari J.R., Leetongin G., Kahlmeier S. (2012). The pandemic of physical inactivity: Global action for public health. Lancet.

[3] World Health Organization Physical Inactivity: A Global Public Health Problem. http://www.who.int/dietphysicalactivity/factsheet_inactivity/en/.

[4] Tremblay M.S., Barnes J.D., Gonzalez S.A., Katzmarzyk P.T., Onywera V.O., Reilly J.J., Tomkinson G.R., Team G.M. (2016). 2 0 R. Global matrix 2.0: Report card grades on the physical activity of children and youth comparing 38 countries. J. Phys. Act. Health.

[5] Tremblay M.S., Willms J.D. (2000). Secular trends in the body mass index of Canadian children. CMAJ.

[6] Canadian Society for Exercise Physiology (2016). Canadian 24-Hour Movement Guidelines and Children and Youth: An Integration of Physical Activity, Sedentary Behaviour, and Sleep.

[7] Roberts K.C., Yao X., Carson V., Chaput J.-P., Janssen I., Tremblay M.S. (2017). Meeting the Canadian 24-hour movement guidelines for children and youth. Health Rep..

[8] Olds T.S., Maher C.A., Ridley K., Kittel D.M. (2010). Descriptive epidemiology of screen and non-screen sedentary time in adolescents: A cross sectional study. Int. J. Behav. Nutr. Phys. Act..

[9] Hinckson E., Cerin E., Mavoa S., Smith M., Badland H., Stewart T., Duncan S., Schofield G. (2017). Associations of the perceived and objective neighborhood environment with physical activity and sedentary time in New Zealand adolescents. Int. J. Behav. Nutr. Phys. Act..

[10] Huang J.-H., Hipp J.A., Marquet O., Alberico C., Fry D., Mazak E., Lovasi G.S., Robinson W.R., Floyd M.F. (2020). Neighborhood characteristics associated with park use and park-based physical activity among children in low-income diverse neighborhoods in New York City. Prev. Med..

[11] Rodríguez D.A., Cho G.-H., Evenson K.R., Conway T.L., Cohen D., Ghosh-Dastidar B., Pickrel J.L., Veblen-Mortenson S., Lytle L.A. (2012). Out and about: Association of the built environment with physical activity behaviors of adolescent females. Health Place.

[12] Rainham D.G., Bates C.J., Blanchard C.M., Dummer T.J., Kirk S.F., Shearer C.L. (2012). Spatial classification of youth physical activity patterns. Am. J. Prev. Med..

[13] McGrath L.J., Hopkins W.G., Hinckson E.A. (2015). Associations of objectively measured built-environment attributes with youth moderate–vigorous physical activity: A systematic review and meta-analysis. Sports Med..

[14] McCormack G.R., Shiell A. (2011). In search of causality: A systematic review of the relationship between the built environment and physical activity among adults. Int. J. Behav. Nutr. Phys. Act..

[15] Prince S.A., Butler G.P., Rao D.P., Thompson W. (2019). Evidence synthesis—Where are children and adults physically active and sedentary?—a rapid review of location-based studies. Health Promot. Chronic Dis. Prev. Can. Res. Policy Pract..

[16] King A.C., Parkinson K.N., Adamson A.J., Murray L., Besson H., Reilly J.J., Basterfield L., Team G.M.S.C. (2011). Correlates of objectively measured physical activity and sedentary behaviour in English children. Eur. J. Public Health.

[17] Pearce M.S., Basterfield L., Mann K.D., Parkinson K.N., Adamson A.J., Reilly J.J., Team G.M.S.C. (2012). Early predictors of objectively measured physical activity and sedentary behaviour in 8–10 year old children: The Gateshead Millennium Study. PLoS ONE.

[18] Fisher A., Reilly J.J., Montgomery C., Kelly L.A., Williamson A., Jackson D.M., Paton J.Y., Grant S. (2005). Seasonality in physical activity and sedentary behavior in young children. Pediatr. Exerc. Sci..

[19] Shen B., Alexander G., Milberger S., Jen K.-L.C. (2013). An exploratory study of seasonality and preschoolers’ physical activity engagement. J. Phys. Act. Health.

[20] Silva P., Santos R., Welk G., Mota J. (2011). Seasonal differences in physical activity and sedentary patterns: The relevance of the PA context. J. Sports Sci. Med..

[21] Hjorth M.F., Chaput J.P., Michaelsen K., Astrup A., Tetens I., Sjodin A. (2013). Seasonal variation in objectively measured physical activity, sedentary time, cardio-respiratory fitness and sleep duration among 8–11 year-old Danish children: A repeated-measures study. BMC Public Health.

[22] Loucaides C.A. (2018). Seasonal differences in segmented-day physical activity and sedentary behaviour in primary school children. Early Child Dev. Care.

[23] Saint-Maurice P., Bai Y., Vazou S., Welk G. (2018). Youth Physical Activity Patterns During School and Out-of-School Time. Children.

[24] Katapally T.R., Rainham D., Muhajarine N. (2015). Factoring in weather variation to capture the influence of urban design and built environment on globally recommended levels of moderate to vigorous physical activity in children. BMJ Open.

[25] Belanger M., Gray-Donald K., O’Loughlin J., Paradis G., Hanley J. (2009). Influence of weather conditions and season on physical activity in adolescents. Ann. Epidemiol..

[26] Rahman S., Maximova K., Carson V., Jhangri G.S., Veugelers P.J. (2019). Stay in or play out? The influence of weather conditions on physical activity of grade 5 children in Canada. Can. J. Public Health.

[27] Colley R.C., Carson V., Garriguet D., Janssen I., Roberts K.C., Tremblay M.S. (2017). Physical activity of Canadian children and youth, 2007 to 2015. Health Rep..

[28] Maitland C., Stratton G., Foster S., Braham R., Rosenberg M. (2013). A place for play? The influence of the home physical environment on children’s physical activity and sedentary behaviour. Int. J. Behav. Nutr. Phys. Act..

[29] Muhajarine N., Katapally T.R., Fuller D., Stanley K.G., Rainham D. (2015). Longitudinal active living research to address physical inactivity and sedentary behaviour in children in transition from preadolescence to adolescence. BMC Public Health.

[30] WHO BMI-for-age (5–19 years). http://www.who.int/growthref/who2007_bmi_for_age/en/.

[31] Heil D.P. (2006). Predicting activity energy expenditure using the Actical^®^ activity monitor. Res. Q. Exerc. Sport.

[32] Esliger D.W., Sherar L.B., Muhajarine N. (2012). Smart cities, healthy kids: The association between neighbourhood design and children’s physical activity and time spent sedentary. Can. J. Public Health Rev. Can. Sante Publique.

[33] Chinapaw M.J., de Niet M., Verloigne M., Bourdeaudhuij I.D., Brug J., Altenburg T.M. (2014). From sedentary time to sedentary patterns: Accelerometer data reduction decisions in youth. PLoS ONE.

[34] Kozey-Keadle S., Libertine A., Lyden K., Staudenmayer J., Freedson P.S. (2011). Validation of wearable monitors for assessing sedentary behavior. Med. Sci. Sports Exerc..

[35] Day K., Boarnet M., Alfonzo M., Forsyth A. (2006). The Irvine-Minnesota Inventory to Measure Built Environments: Development. Am. J. Prev. Med..

[36] Gauvin L., Richard L., Craig C.L., Spivock M., Riva M., Forster M., Laforest S., Laberge S., Fournel M.-C., Gagnon H. (2005). From walkability to active living potential: An “ecometric” validation study. Am. J. Prev. Med..

[37] Boarnet M.G., Day K., Alfonzo M., Forsyth A., Oakes M. (2006). The Irvine-Minnesota inventory to measure built environments: Reliability tests. Am. J. Prev. Med..

[38] Fuller D.L., Muhajarine N. (2010). Replication of the neighborhood active living potential measure in Saskatoon, Canada. Am. J. Prev. Med..

[39] Gauvin L., Riva M., Barnett T., Richard L., Craig C.L., Spivock M., Laforest S., Laberge S., Fournel M.C., Gagnon H. (2008). Association between neighborhood active living potential and walking. Am. J. Epidemiol..

[40] Boarnet M.G., Forsyth A., Day K., Oakes J.M. (2011). The street level built environment and physical activity and walking: Results of a predictive validity study for the Irvine Minnesota Inventory. Environ. Behav..

[41] Defining Seasons. https://www.timeanddate.com/calendar/aboutseasons.html.

[42] R Core Team (2018). R: A Language and Environment for Statistical Computing.

[43] RStudio Team (2016). RStudio: Integrated Development Environment for R.

[44] van Rossum G., de Boer J. (1991). Interactively testing remote servers using the Python programming language. CWi Q..

[45] QGIS Development Team QGIS Geographic Information System [Internet]. Open Source Geospatial Foundation Project..

[46] Statistics Canada (2017). Canada [Country] and Saskatoon, CY [Census subdivision], Saskatchewan (table). Census Profile. 2016 Census. [Internet].

[47] Modupalli K., Cushon J., Neudorf C. (2013). 2010/2011 Student Health Survey: Evidence for Action.

[48] Statistics Canada Table 13-10-0797-01 Measured Children and Youth Body Mass Index (BMI) (Cole Classification), by Age Group and Sex, Canada and Provinces, Canadian Community Health Survey—Nutrition. https://www150.statcan.gc.ca/t1/tbl1/en/tv.action?pid=1310079701.

[49] Statistics Canada (2017). Immigrant Status and Period of Immigration (11), Place of Birth (272), Age (7A) and Sex (3) for the Population in Private Households of Canada, Provinces and Territories, Census Divisions and Census Subdivisions, 2016 Census—25% Sample Data.

[50] Sallis J.F., Prochaska J.J., Taylor W.C. (2000). A review of correlates of physical activity of children and adolescents. Med. Sci. Sports Exerc..

[51] Sallis J.F., Taylor W.C., Dowda M., Freedson P.S., Pate R.R. (2002). Correlates of vigorous physical activity for children in grades 1 through 12: Comparing parent-reported and objectively measured physical activity. Pediatr. Exerc. Sci..

[52] Dumith S.C., Gigante D.P., Domingues M.R., Kohl H.W. (2011). Physical activity change during adolescence: A systematic review and a pooled analysis. Int. J. Epidemiol..

[53] Sallis J.F. (2000). Age-related decline in physical activity: A synthesis of human and animal studies. Med. Sci. Sports Exerc..

[54] Cairney J., Veldhuizen S., Kwan M., Hay J., Faught B.E. (2014). Biological age and sex-related declines in physical activity during adolescence. Med. Sci. Sports Exerc..

[55] Kristjansdottir G., Vilhjálmsson R. (2001). Sociodemographic differences in patterns of sedentary and physically active behavior in older children and adolescents. Acta Paediatr..

[56] Bacil E.D.A., Piola T.S., Watanabe P.I., da Silva M.P., Legnani R.F.S., da Campos W., Bacil E.D.A., Piola T.S., Watanabe P.I., da Silva M.P. (2016). Biological maturation and sedentary behaviour in children and adolescents: A systematic review. J. Phys. Educ..

[57] Stone M.R., Faulkner G.E., Mitra R., Buliung R.N. (2014). The freedom to explore: Examining the influence of independent mobility on weekday, weekend and after-school physical activity behaviour in children living in urban and inner-suburban neighbourhoods of varying socioeconomic status. Int. J. Behav. Nutr. Phys. Act..

[58] Mitra R., Faulkner G.E., Buliung R.N., Stone M.R. (2014). Do parental perceptions of the neighbourhood environment influence children’s independent mobility? Evidence from Toronto, Canada. Urban Stud..

[59] He M., Piché L., Beynon C., Harris S. (2010). Screen-related sedentary behaviours: Children’s and parents’ attitudes, motivations, and practices. J. Nutr. Educ. Behav..

[60] Sisson S.B., Broyles S.T., Baker B.L., Katzmarzyk P.T. (2011). Television, reading, and computer time: Correlates of school-day leisure-time sedentary behavior and relationship with overweight in children in the U.S. J. Phys. Act. Health.

[61] Liou Y.M., Liou T.-H., Chang L.-C. (2010). Obesity among adolescents: Sedentary leisure time and sleeping as determinants. J. Adv. Nurs..

[62] Katapally T.R., Laxer R.E., Qian W., Leatherdale S.T. (2018). Do school physical activity policies and programs have a role in decreasing multiple screen time behaviours among youth?. Prev. Med..

[63] Veitch J., Salmon J., Ball K. (2008). Children’s active free play in local neighborhoods: A behavioral mapping study. Health Educ. Res..

[64] Shearer C., Blanchard C., Kirk S., Lyons R., Dummer T., Pitter R., Rainham D., Rehman L., Shields C., Sim M. (2012). Physical activity and nutrition among youth in rural, suburban and urban neighbourhood types. Can. J. Public Health Rev. Can. Sante Publique.

[65] Loucaides C.A., Chedzoy S.M., Bennett N. (2004). Differences in physical activity levels between urban and rural school children in Cyprus. Health Educ. Res..

[66] Williams G.C., Borghese M.M., Janssen I. (2018). Neighborhood walkability and objectively measured active transportation among 10–13 year olds. J. Transp. Health.

[67] Demant Klinker C., Schipperijn J., Toftager M., Kerr J., Troelsen J. (2015). When cities move children: Development of a new methodology to assess context-specific physical activity behaviour among children and adolescents using accelerometers and GPS. Health Place.

